# Characterization of simian T-cell leukemia virus type 1 in naturally infected Japanese macaques as a model of HTLV-1 infection

**DOI:** 10.1186/1742-4690-10-118

**Published:** 2013-10-24

**Authors:** Michi Miura, Jun-ichiro Yasunaga, Junko Tanabe, Kenji Sugata, Tiejun Zhao, Guangyong Ma, Paola Miyazato, Koichi Ohshima, Akihisa Kaneko, Akino Watanabe, Akatsuki Saito, Hirofumi Akari, Masao Matsuoka

**Affiliations:** 1 Laboratory of Virus Control, Institute for Virus Research, Kyoto University, Shogoin Kawahara-cho 53, Sakyo-ku, Kyoto 606-8507, Japan; 2 Department of Pathology, School of Medicine, Kurume University, Kurume, Fukuoka, Japan; 3 Center for Human Evolution Modeling Research, Primate Research Institute, Kyoto University, Inuyama, Aichi, Japan; 4 Present address: College of Chemistry and Life Sciences, Zhejiang Normal University, Jinhua, China

**Keywords:** Simian T-cell leukemia virus, Human T-cell leukemia virus, Tax, HBZ

## Abstract

**Background:**

Human T-cell leukemia virus type 1 (HTLV-1) causes chronic infection leading to development of adult T-cell leukemia (ATL) and inflammatory diseases. Non-human primates infected with simian T-cell leukemia virus type 1 (STLV-1) are considered to constitute a suitable animal model for HTLV-1 research. However, the function of the regulatory and accessory genes of STLV-1 has not been analyzed in detail. In this study, STLV-1 in naturally infected Japanese macaques was analyzed.

**Results:**

We identified spliced transcripts of STLV-1 corresponding to HTLV-1 *tax* and HTLV-1 bZIP factor (*HBZ*). STLV-1 Tax activated the NFAT, AP-1 and NF-κB signaling pathways, whereas STLV-1 bZIP factor (SBZ) suppressed them. Conversely, SBZ enhanced TGF-β signaling and induced Foxp3 expression. Furthermore, STLV-1 Tax activated the canonical Wnt pathway while SBZ suppressed it. STLV-1 Tax enhanced the viral promoter activity while SBZ suppressed its activation. Then we addressed the clonal proliferation of STLV-1^+^ cells by massively sequencing the provirus integration sites. Some clones proliferated distinctively in monkeys with higher STLV-1 proviral loads. Notably, one of the monkeys surveyed in this study developed T-cell lymphoma in the brain; STLV-1 provirus was integrated in the lymphoma cell genome. When anti-CCR4 antibody, mogamulizumab, was administered into STLV-1-infected monkeys, the proviral load decreased dramatically within 2 weeks. We observed that some abundant clones recovered after discontinuation of mogamulizumab administration.

**Conclusions:**

STLV-1 Tax and SBZ have functions similar to those of their counterparts in HTLV-1. This study demonstrates that Japanese macaques naturally infected with STLV-1 resemble HTLV-1 carriers and are a suitable model for the investigation of persistent HTLV-1 infection and asymptomatic HTLV-1 carrier state. Using these animals, we verified that mogamulizumab, which is currently used as a drug for relapsed ATL, is also effective in reducing the proviral load in asymptomatic individuals.

## Background

Human T-cell leukemia virus type 1 (HTLV-1) was the first human retrovirus found to cause a neoplastic disease, adult T-cell leukemia (ATL) [1,2]. Approximately 10 million people worldwide are estimated to be infected with this virus. HTLV-1 is endemic in specific areas including southwestern Japan, Central and South America, the Caribbean, and intertropical Africa [3]. Most HTLV-1 carriers remain asymptomatic through their lives and only a small fraction of them develop ATL, a leukemia of HTLV-1-infected CD4^+^ T cells, after a long latent period [4]. This virus also causes inflammatory disorders such as HTLV-1-associated myelopathy/tropic spastic paraparesis (HAM/TSP) [5,6] and uveitis [7].

The reason why most HTLV-1 carriers do not develop ATL is partly explained by the immune response of cytotoxic T cells (CTLs) against HTLV-1 proteins [8]. Immunosuppressive conditions, particularly following organ or bone marrow transplantation, can induce the development of ATL [9,10], indicating that the host immune system usually prevents the development of ATL. Two HTLV-1 proteins, Tax and HTLV-1 bZIP factor (HBZ), are thought to promote the proliferation of infected cells and ATL cells [4,11]. Tax is highly immunogenic to CTLs and the infected cells expressing Tax are kept to a small number [12]. Recently, it has been reported that CTLs to HBZ play a critical role in determining proviral load in carriers [13].

Animal models that are relevant to the human immune system are required for scientists to investigate how the immune response controls the proliferation of infected cells and viral replication *in vivo*. Old World monkeys are frequently infected with simian T-cell leukemia virus type 1 (STLV-1), which is closely related to HTLV-1 [14]. Like HTLV-1 infection, clonal proliferation of STLV-1-infected cells was detected by inverse PCR [15]. Furthermore, STLV-1 also leads to the development of lymphoproliferative diseases [16,17]. Based on these observations, it has been proposed that STLV-1-infected non-human primates may constitute a suitable animal model for HTLV-1 research. However, a detailed characterization of STLV-1 infection in non-human primates has not been achieved.

In the present study, Japanese macaques naturally infected with STLV-1 were investigated. We first identified the STLV-1 bZIP factor (SBZ) gene as an antisense transcript of STLV-1 similar to HBZ. Molecular analyses showed that STLV-1 Tax and SBZ have activities on various transcriptional pathways similar to those of HTLV-1 Tax and HBZ. Furthermore, we observed clonal proliferation of STLV-1-infected cells. Finally, anti-CCR4 antibody, which is currently used to treat ATL patients, was administered into STLV-1-infected Japanese macaques, and we found that this reduced the proviral load *in vivo*, indicating that anti-CCR4 antibody is effective for treatment of HTLV-1-associated inflammatory diseases. These results suggest that Japanese macaques naturally infected with STLV-1 show characteristics that correlate closely with those of HTLV-1 carriers and may therefore serve as a suitable animal model for the analysis of persistent HTLV-1 infection and HTLV-1 carrier state.

## Results

### Seroprevalence and proviral load of STLV-1 in Japanese macaques

**Figure 1:**
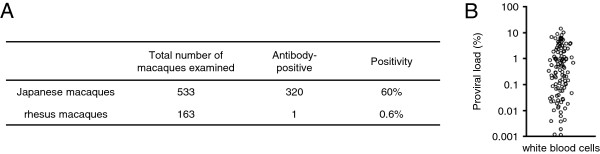
**STLV-1 infection in Japanese and rhesus macaques. (A)** STLV-1 seropositivity in Japanese macaques and rhesus macaques screened in this study is shown. **(B)** STLV-1 proviral load (percentage) in white blood cells of Japanese macaques is shown.

To identify STLV-1-infected monkeys, we screened plasma samples for antibody against viral STLV-1 antigens by particle-agglutination test. Out of 533 Japanese macaques examined, 320 (60%) were seropositive, while only one rhesus macaque out of 163 (0.6%) was seropositive (Figure 1A). Proviral load in white blood cells was measured by quantitative real-time PCR for 115 seropositive Japanese macaques. Proviral load ranged from 0.001% to over 10% (Figure 1B). Since the DNA samples used in the above experiment were obtained from total white blood cells including granulocytes, these data likely underestimate proviral load of PBMCs.

### Functional similarity of STLV-1 Tax and STLV-1 bZIP factor to their counterparts in HTLV-1

**Figure 2:**
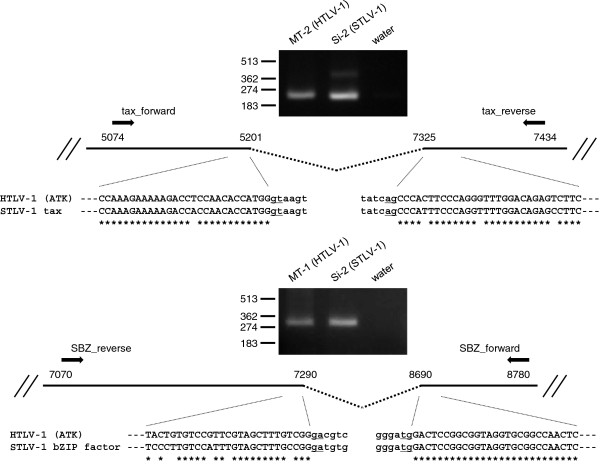
**Detection of STLV-1 *tax *and *STLV-1 bZIP factor *(*SBZ*) transcripts and their splicing junctions.** STLV-1 *tax* and *SBZ* transcripts were amplified by RT-PCR using the primers flanking the putative splicing site. The bands of the amplified fragments are shown together with the corresponding transcript of HTLV-1 in the images of agarose gel stained with ethidium bromide. Numbers in the scheme indicate the nucleotide positions of HTLV-1 ATK provirus. Sequences of the amplified STLV-1 *tax* and *SBZ* transcripts are represented with uppercase letters and aligned with a reference sequence of HTLV-1 (ATK). The lowercase letters represent the intron region of HTLV-1 or STLV-1 provirus.

**Figure 3:**
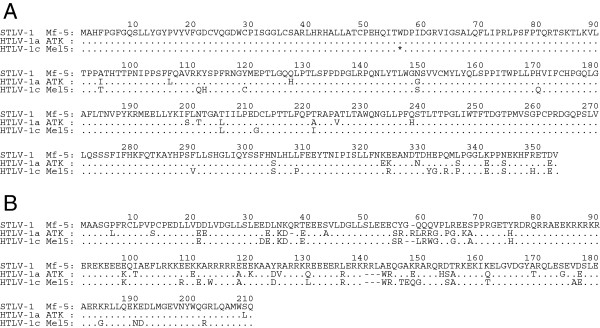
**Comparison of the amino acid sequences of STLV-1 Tax and SBZ with those of HTLV-1 Tax and HBZ.** Amino acid sequences of STLV-1 Tax **(A)** and SBZ **(B)** derived from an STLV-1^+^ Japanese macaque (Mf-5) are compared respectively with those of HTLV-1 Tax and HBZ from two isolates. Asterisk represents the termination codon. Accession number: [GenBank:J02029] (ATK) and [GenBank:L02534] (Mel5).

Analysis of the STLV-1 pX region suggests the presence of *tax* coding gene and an antisense transcript in the minus strand of STLV-1 similar to *HBZ*. In order to examine if STLV-1 *tax* and *SBZ* genes are transcribed and processed to be mature mRNAs in STLV-1-infected PBMCs, STLV-1 *tax* and *SBZ* transcripts were amplified by RT-PCR using the primers flanking the putative splicing site (Figure 2). The length of the amplified fragments was comparable to that of the corresponding HTLV-1 transcripts, which are approximately 240 bp for *tax* and 310 bp for *HBZ*. We further verified that STLV-1 *tax* and *SBZ* transcripts are spliced at exactly the same location as HTLV-1 *tax* and spliced form of *HBZ*[11,18], respectively (Figure 2). To investigate the molecular functions of STLV-1 Tax and SBZ, we cloned the coding sequences of those proteins from the STLV-1 provirus in a Japanese macaque (Mf-5). Approximately 91% of the coding sequence of *tax* was identical in HTLV-1 (ATK) and Japanese macaque STLV-1, and 82% in *HBZ* (ATK) and Japanese macaque *SBZ*. Phylogenetic analyses show that Japanese macaque STLV-1 *env* in this study is close to Melanesian subtype C [5] (Additional file 1). Therefore, the STLV-1 protein sequences were aligned with HTLV-1 prototype ATK (subtype A) as well as Mel5 (subtype C) for comparison, and presented in Figure 3. Approximately 93% of the STLV-1 Tax amino acid sequence was identical to that of HTLV-1 Tax (Figure 3A) and approximately 73% of the amino acid sequence of SBZ was identical to that of HBZ (Figure 3B). Notably, SBZ has some insertions and deletions, resulting in an excess of three amino acids compared with HBZ.

**Figure 4:**
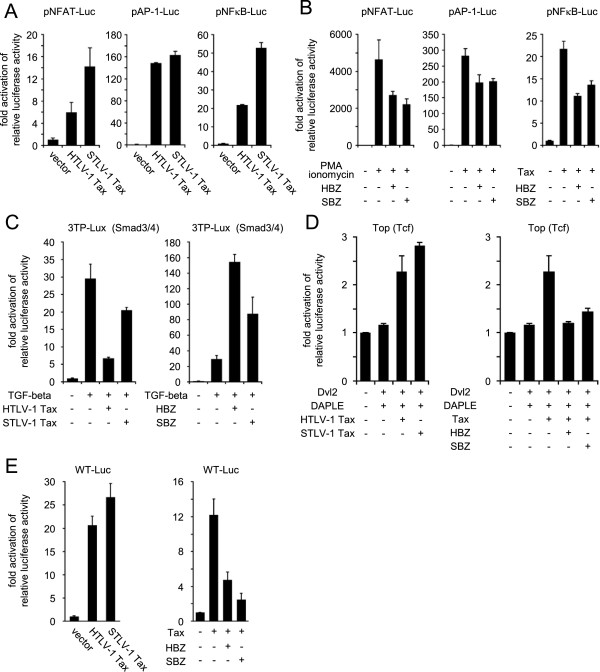
**Effects of STLV-1 Tax and SBZ on various signaling pathways.** Effects of HTLV-1 Tax or STLV-1 Tax **(A)**, and HBZ or SBZ **(B)** were analyzed using reporter plasmids for the NFAT, AP-1 and NF-κB pathways in Jurkat cells. **(C)** The effects of HTLV-1 Tax or STLV-1 Tax (left) and HBZ or SBZ (right) on the TGF-β signaling pathway were analyzed in HepG2 cells using the reporter plasmid 3TP-Lux, which contains the responsive element to Smad3/4. **(D)** The effects of HTLV-1 Tax or STLV-1 Tax (left) and HBZ or SBZ (right) on relative luciferase activity driven by TCF-responsive elements were analyzed using Jurkat cells. **(E)** The effects of HTLV-1 Tax or STLV-1 Tax (left) and HBZ or SBZ (right) on relative luciferase activity driven by viral LTR were analyzed using Jurkat cells. Firefly luciferase activity was normalized to that of Renilla luciferase and represented as fold activation compared to the relevant control. The data represent mean and standard deviation.

It was previously shown that HTLV-1 Tax activates the NF-κB, NFAT and AP-1 pathways [19,20], whereas HBZ suppresses them [21]. The effect of STLV-1 Tax on these pathways was analyzed using luciferase assays. We found that, like HTLV-1 Tax, STLV-1 Tax activated these pathways (Figure 4A). Conversely, SBZ suppressed these pathways when they were activated by phorbol myristate acetate and ionomycin (NFAT and AP-1) or HTLV-1 Tax (NF-κB) (Figure 4B).

**Figure 5:**
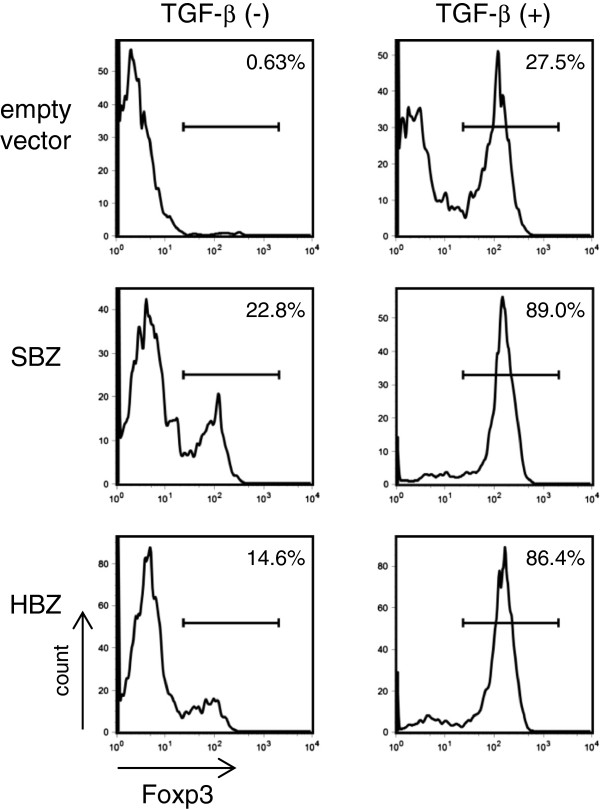
**Flow cytometric analyses of Foxp3 induction by SBZ.** SBZ or HBZ transduced mouse T cells that were positive for the transduction marker were analyzed for Foxp3 expression. The percentage of cells positive for Foxp3 is shown in each histogram. Each experiment was done at least in triplicate, and representative results are shown.

Recently, our group reported that HBZ enhances TGF-β signaling via interaction with Smad2/3 and p300, thus inducing the expression of Foxp3 *in vitro*[22]. The analysis of HBZ transgenic mice further demonstrated an increase in Foxp3^+^ T cells [23]. Therefore, we investigated whether SBZ also enhances TGF-β signaling. We found that SBZ enhanced signaling by the TGF-β pathway, while STLV-1 Tax suppressed it (Figure 4C). Like HBZ, expression of SBZ in mouse naïve CD4^+^ T cells induced expression of Foxp3, and this expression was significantly enhanced by TGF-β (Figure 5). Thus, SBZ, like its counterpart HBZ, activates the TGF-β/Smad pathway and induces Foxp3 expression in CD4^+^ T cells.

Next we studied STLV-1 Tax and SBZ for their capability to regulate the canonical Wnt pathway in the manner we recently reported for HTLV-1 Tax and HBZ [24]. STLV-1 Tax, like HTLV-1 Tax, elevated the activity of luciferase regulated by the promoter responsive to TCF/LEF in the presence of Dvl2 and DAPLE (Figure 4D). In contrast, when SBZ was co-expressed with Tax, luciferase activity was suppressed (Figure 4D). These results demonstrate that like their counterparts in HTLV-1, STLV-1 Tax activates the canonical Wnt pathway while SBZ suppresses it.

Lastly, regulation of viral promoter activity by STLV-1 Tax and SBZ was examined since it is known that HTLV-1 Tax activates the viral transcription from the 5’ long terminal repeat (LTR) of the provirus while HBZ suppresses it. As presented in Figure 4E, STLV-1 Tax activated transcription of WT-Luc while SBZ suppressed it in Jurkat cells. It is consistent with functions of HTLV-1 Tax and HBZ.

### Clonal proliferation of STLV-1-infected cells in Japanese macaques

**Figure 6:**
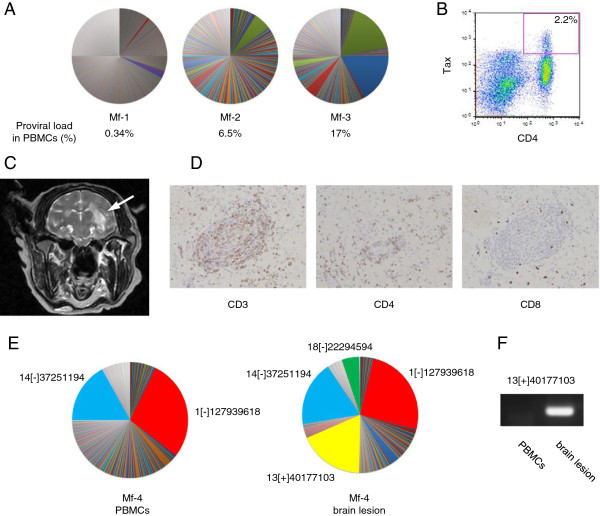
**Clonal proliferation of STLV-1-infected cells and lymphomatous lesion in the STLV-1-infected Japanese macaque. (A)** The relative frequency of STLV-1^+^ clones in three monkeys (Mf-1, Mf-2 and Mf-3) is presented. Each area in the pie charts represents the proportion of provirus in a separate clone (identified by its unique integration site). **(B)** Flow cytometric analysis of PBMCs from an STLV-1-infected monkey shows that Tax-expressing cells are positive for CD4. **(C)** Magnetic resonance imaging of the brain of monkey Mf-4. The lesion is indicated by the white arrow. **(D)** Immunohistochemical analyses show that lymphoma cells are positive for CD3 and CD4. **(E)** Relative abundance of STLV-1^+^ clones identified by unique integration sites of the provirus in PBMCs (left) and in the brain lesion (right) of Mf-4. Some of the abundant clones that are observed both in PBMCs and the brain lesion are painted in the same color in the two pie charts. **(F)** STLV-1^+^ abundant clone 13[+]40177103 is detected in the brain lesion by using the primers for 3’ LTR and the genomic region, but not in PBMCs.

Clonal proliferation of HTLV-1-infected cells has been demonstrated by inverse PCR and next generation sequencing methods [25-27]. We analyzed the clonality of STLV-1-infected cells in seropositive Japanese macaques by identifying the genomic sequences adjacent to the 3’ LTR. Briefly, genomic DNAs of monkey PBMCs were sheared by sonication and the integration sites of the provirus adjacent to the viral 3’ LTR were amplified by linker-mediated PCR. Thereafter, we massively sequenced the integration sites and analyzed the abundance of each clones according to the method reported by Gillet et al. [27]. The detailed information on the deep sequencing is described in Additional file 2. The clonality of STLV-1-infected cells in three monkeys is shown in Figure 6A. Proviral load is represented as the percentage of STLV-1-infected cells in PBMCs. In monkeys with lower proviral load, a few major clones, together with many minor ones, were observed in Mf-1. Some clones proliferated in Mf-2 (Figure 6A, left and middle). On the other hand, another monkey, Mf-3, which had higher proviral load (17%), possessed two major STLV-1-infected clones (Figure 6A, right). To study which cell types are infected by STLV-1, Tax expression in PBMCs obtained from one monkey (Mf-4) was analyzed by flow cytometry. The Tax-expressing cells were largely found to be CD4^+^ T cells, as is the case with HTLV-1 infection in humans (Figure 6B).

### STLV-1-associated T-cell lymphoma in a Japanese macaque

A monkey (Mf-4) developed anorexia and had paralysis of the lower limbs. This monkey had high proviral load (53%) in PBMCs. We suspected that this monkey has developed a disease similar to HAM/TSP because paralysis of the lower limbs is one of the major symptoms of HAM/TSP patients. Magnetic resonance imaging (MRI) revealed a high intensity lesion in the brain on a T2-weighted image (Figure 6C). Pathological analysis showed that this tumor was a lymphoma with atypical morphology, and by immunohistochemical methods, it was found that these cells were CD3^+^ CD4^+^ (Figure 6D). In contrast, no obvious demyelination was observed in the spinal cord. Thus, this monkey was diagnosed with T-cell lymphoma in the brain rather than the disease like HAM/TSP. In this monkey, some major clones had proliferated in peripheral blood (Figure 6E, left). We found that the major clones in peripheral blood were also detected in the brain lesion (Figure 6E, right). These observations demonstrate that STLV-1 causes lymphoma in Japanese macaques. Notably, one of the major clones in the brain, which had its provirus integration site in chromosome 13, was not detected in PBMCs. This was confirmed by conventional PCR using the primers for the 3’LTR and the host genome proximal to the integration site (Figure 6F). Moreover, a clone with the integration site in chromosome 18 was also detected only in the brain lesion. These tumor-specific STLV-1-infected clones are thought to contribute to the formation of the tumor.

### Treatment with anti-CCR4 antibody decreased proviral load in STLV-1-infected Japanese macaques

**Figure 7:**
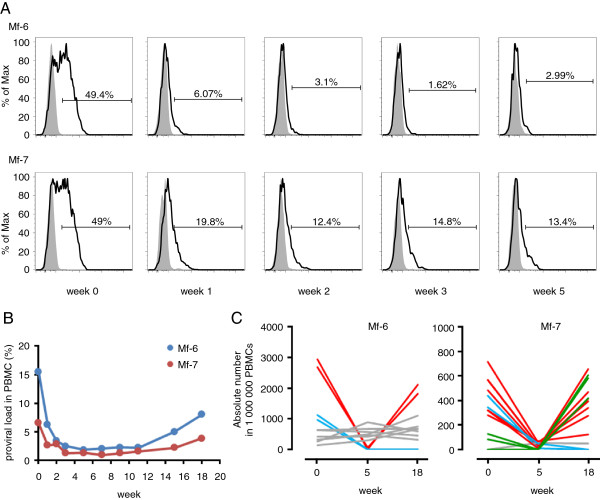
**Effect of anti-CCR4 antibody on STLV-1 dynamics *in vivo*. (A)** CD3^+^CD4^+^ T cells were gated and the expression of CCR4 was analyzed by flow cytometry. **(B)** Changes in STLV-1 proviral load in two monkeys treated with anti-CCR4 antibody until week 3. **(C)** Absolute cell numbers of the five most abundant clones in 1,000,000 PBMCs at weeks 0, 5 and 18 are shown.

ATL cells express high levels of CC chemokine receptor 4 (CCR4) [28]. Recently, mogamulizumab, a humanized IgG1 monoclonal antibody against CCR4 [29], was approved in Japan for the treatment of relapsed ATL patients. HTLV-1-infected cells of healthy carriers also express CCR4, which indicates that mogamulizumab likely reduces the proviral load in HTLV-1-infected asymptomatic individuals [30]. High proviral load has been reported to be associated with HAM/TSP, HTLV-1 uveitis, and risk of ATL, indicating that mogamulizumab may potentially be used for the treatment of HTLV-1-associated diseases and the prevention of ATL. However, it is not clear whether mogamulizumab can reduce the proviral load in HTLV-1-infected individuals. We confirmed that mogamulizumab also recognizes macaque CCR4 by staining Japanese macaque PBMCs *in vitro* with the fluorescently labeled antibody (see Additional file 3). Then, we tested the efficacy of mogamulizumab to reduce the proviral load in STLV-1-infected Japanese macaques. Mogamulizumab was administered to two monkeys with high proviral load (Mf-6 and Mf-7), once a week for 4 weeks. As shown in Figure 7A, nearly half of the CD4^+^ T cells expressed CCR4 before the treatment (week 0). After the treatment, the CCR4 positivity decreased to 1.62% and 12.4% respectively. We also measured proviral load over the course of the treatment and found that it decreased dramatically within 2 weeks (Figure 7B). Thus, this demonstrates that mogamulizumab can indeed reduce the number of STLV-1-infected cells *in vivo*.

Eight weeks after the final administration of mogamulizumab, the proviral load started to recover (Figure 7B). To investigate whether mogamulizumab influences the clonality of STLV-1-infected cells, we evaluated the absolute number of each clone by high-throughput sequencing of provirus integration sites. Figure 7C shows changes of the five most abundant clones at weeks 0, 5 and 18. The major clones before the treatment (week 0) recovered at week 18 (red lines in Figure 7C), while some clones were present constantly during the treatment (grey lines) or diminished after the treatment (blue lines). Interestingly, some clones (green lines) that emerged in a monkey after treatment were rare or even not detected before treatment (Figure 7C).

## Discussion

HTLV-1 is thought to originate from STLV-1. In STLV-1-infected monkeys, investigators found clonal proliferation of STLV-1-infected cells and the preferential infection of CD4^+^ T cells by the virus [15,31]. Moreover, several groups reported the development of lymphomas in STLV-1-infected monkeys [16,17,32-35]. Monoclonal integration of STLV-1 in the lymphoproliferative disease of African green monkeys was detected by Southern blot [16,33], demonstrating the direct causative role of STLV-1. Thus STLV-1-infected non-human primates have been thought to be a useful animal model for HTLV-1 research. The dynamics of infected cells after treatment with histone deacetylase inhibitors and reverse transcriptase inhibitors has been analyzed in STLV-1-infected baboons, and it was found that this combination significantly decreased proviral load in treated animals [36]. However, there have been no detailed studies on functions of STLV-1-encoded genes. Analyses of the functions of its accessory and regulatory proteins are necessary if we are to use STLV-1-infected monkeys as a model of HTLV-1 infection. In the present study, we focused on Japanese macaques naturally infected with STLV-1.

The amino acid sequence of STLV-1 Tax is closely homologous to that of HTLV-1 Tax, and this study demonstrated that their functions on various transcriptional pathways are similar as well. This study was the first to identify SBZ as an antisense transcript of STLV-1 and a homolog of HBZ. SBZ and HBZ share only approximately 73% identity at the amino acid level. Nevertheless, for all the functions we examined, SBZ behaves similarly to HBZ. In particular, SBZ expression could induce Foxp3 expression like HBZ expression does. This might be attributed to the following reasons. First, the N-terminal region, as well as the heptad repeats of hydrophobic amino acids in the basic leucine zipper domain, are conserved between HBZ and SBZ. This may allow SBZ to interact with and suppress NF-κB, AP-1 and other transcription factors with basic leucine zipper motifs [37,38]. Second, the LXXLL-like region (Leu27, Leu28, Leu48 and Leu49), which is critical for the interaction with p300 and Smad3 protein, is also conserved between HBZ and SBZ [22,39]. Some lysine residues present in HBZ are substituted with different amino acids in Japanese macaque SBZ. This study showed that SBZ has similar functions compared with HBZ, suggesting that these lysine residues are not critical for their functions. However, further studies are necessary for deep understanding of implication of these amino acid sequences.

HTLV-1 increases the number of infected cells by clonal proliferation of infected cells, which likely facilitates cell-to-cell transmission of this virus. Clonal proliferation of STLV-1-infected cells in Celebes macaques was demonstrated by the conventional inverse PCR method [15]. However, this technique could detect only a limited population of the clones because of its limited sensitivity or the stochastic amplification of the integration sites. In the present study, we investigated more comprehensively the clonal proliferation of infected cells in Japanese macaques naturally infected with STLV-1 by massively sequencing the unique integration sites of the provirus. The finding that STLV-1-infected cells proliferated clonally in the monkeys with higher proviral loads resembles the finding for HTLV-1. Furthermore, one monkey had lymphoma in the brain, showing that STLV-1 induces lymphoma in Japanese macaques. Analyses of STLV-1 integration sites in this T-cell lymphoma showed that one of the major clones in the brain was unique to this tumor, suggesting that this clone played an important role in the lymphomagenesis of this tumor.

This study also revealed a remarkable difference in STLV-1 seroprevalence between Japanese macaques (320/533: 60%) and rhesus macaques (1/163: 0.6%). Previous studies showed that the seroprevalence in rhesus macaques was 25%, and that in Japanese macaques was quite high [40-42]. Similarly, high seroprevalence was reported in baboons [43]. Furthermore, many studies reported the development of lymphoma in baboons [17,44,45]. The high seroprevalence and the development of lymphomas in Japanese macaques and baboons may suggest a higher susceptibility of these species to STLV-1 infection. Japanese macaques and baboons infected with STLV-1 may be suitable models for HTLV-1 research.

In this study, we also demonstrated that mogamulizumab strongly suppressed proviral load in STLV-1-infected Japanese macaques. Proviral load was suppressed for 4 weeks after the final administration of mogamulizumab, which seems reasonable when considering that the half-life of the antibody administered at 1.0 mg/kg is approximately 18 days as measured in a clinical trial [46]. Some STLV-1-infected major clones recovered after the treatment, while other clones were still suppressed or even not detected. In HTLV-1-infected individuals, HTLV-1 proviral load is relatively constant in the chronic phase, although some minor clones fluctuate [25]. This study is the first to report that most of the major clones recover after the withdrawal of mogamulizumab. This observation suggests that the major clones may have some growth advantages that allow them to proliferate robustly *in vivo*. These growth advantages may be due to the integration site of the provirus, accumulation of genetic mutations, or epigenetic changes. The population of some clones remained constant over the course of the treatment. We speculate that these clones are negative for CCR4 expression. High proviral load is associated with risk of ATL and inflammatory diseases. Therefore, suppression of proviral load by mogamulizumab is a possible treatment for HTLV-1-associated inflammatory diseases such as HAM/TSP.

## Conclusions

In summary, this study is the first to show that STLV-1 Tax and SBZ have activities similar to those of Tax and HBZ, activities which likely induce clonal proliferation and T-cell lymphoma in infected monkeys. STLV-1-infected Japanese macaques appear to be a good model for studying the effects of anti-viral drugs and the immunological aspects of HTLV-1 infection.

## Methods

### Biological samples of macaques

Japanese macaques (*Macaca fuscata*) and rhesus macaques (*Macaca mulatta*) used in this study were reared in the Primate Research Institute, Kyoto University. Blood samples were obtained from the macaques (for routine veterinary and microbiological examination) under ketamine anesthesia. All animal studies were conducted in accordance with the protocols of experimental procedures that were approved (2011–095) by the Animal Welfare and Animal Care Committee of the Primate Research Institute of Kyoto University, Inuyama, Japan.

### Antibody screening and measurement of proviral load

Plasma samples were screened for the presence of antibodies against HTLV-1 by particle-agglutination test using SERODIA-HTLV-1 (Fujirebio). Proviral load was measured by real-time PCR quantifying the copy number of *tax* and *RAG1* as previously described [47]. Primers and probes are available in Additional file 4.

### Detection of STLV-1 transcripts

Total RNA was extracted from STLV-1-infected Japanese macaque cell line Si-2 [48] with Trizol (Invitrogen), then cDNA was synthesized with SuperScript III (Invitrogen) using oligo dT primer. STLV-1 *tax* and *SBZ* was detected by PCR using primers (see Additional file 4) from the synthesized Si-2 cDNA: for STLV-1 *tax*, 2 min at 95°C, followed by 35 cycles of 20 seconds at 95°C, 10 seconds at 61°C, and 30 seconds at 72°C, and additional 5 min at 72°C; for SBZ, 2 min at 95°C, followed by 35 cycles of 20 seconds at 95°C, 10 seconds at 58°C, and 30 seconds at 72°C, and additional 5 min at 72°C. For comparison, HTLV-1 *tax* and *HBZ* were also amplified by PCR using cDNA of HTLV-1-infected cell lines (MT-1 or MT-2) with the same conditions. The primers used are shown in Additional file 4.

### Plasmids

The PathDetect pNFκB-Luc, pAP-1-Luc and pNFAT-Luc plasmids were purchased from Stratagene. The 3TP-Lux, TopFlash reporter plasmids and WT-Luc were described previously [22,49]. The coding sequences of STLV-1 Tax and SBZ were amplified from STLV-1 provirus using oligos (see Additional file 4) and cloned into pME18Sneo to generate expression plasmids of STLV-1 Tax and SBZ. HTLV-1 tax was amplified using flanking primers (see Additional file 4) from pCGTax [50] and subcloned into pME18Sneo. The expression vector of HBZ cloned into pME18Sneo was described previously [11]. For the reporter assay, Jurkat cells or HepG2 cells were co-transfected with the reporter plasmid and the viral protein expression plasmids specified in each experiment, as previously described [22,24,51]. The activity of firefly luciferase was represented by normalizing to that of Renilla luciferase.

### Retroviral vectors

The SBZ coding fragment was inserted into pGCDNSamI/N utilizing the NotI and SalI sites and SBZ-expressing retroviral vector was prepared as described previously [22].

### Transduction of primary T-cells with retroviral vectors

CD4^+^CD25^-^ mouse T lymphocytes were stimulated and transduced with SBZ-expressing retroviral vector as previously described [22]. Forty-eight hours after the transduction, cells were harvested and analyzed by flow cytometry.

### Flow cytometry

Antibodies used in this study were as follows: anti-human CD4 (OKT4), anti-Tax MI-73 [52], anti-mouse CD4 (RM4-5), anti-human CD271 (NGFR) (C40-1457), anti-mouse Foxp3 (FJK-16s), anti-human CD3 (SP34-2) and anti-human CCR4 (1G1, which recognizes a different epitope from that recognized by mogamulizumab). Intracellular staining was performed as previously described for Tax [52] and Foxp3 [22]. Cells were analyzed by BD FACSCanto II with FACS Diva Software (BD Biosciences) or BD FACSVerse with FACSuite software (BD Biosciences).

### Deep sequencing of provirus integration sites

The provirus integration sites in the Japanese macaque genome were amplified by linker-mediated PCR as previously described [27], with some modifications. Japanese macaque PBMC genomic DNA (3 μg) was sheared by sonication with a Bioruptor UCD-200 TM to obtain DNA fragments of approximately 200–500 bp. The ends of the DNA fragments were repaired to generate blunt ends using 18 units of T4 DNA polymerase, 5.3 units of DNA Klenow Polymerase I and 18 units of T4 polynucleotide kinase (TOYOBO) in T4 DNA ligase buffer (NEB) supplemented with 300 μM each of dNTP (TAKARA Bio). Adenine nucleotides were added to the blunt ends, and then linkers were ligated using 24 units of T4 DNA ligase (TOYOBO) in T4 DNA ligase buffer (NEB) utilizing the overhang of one thymidine nucleotide at the 3’ end of the linker. The linker was generated by annealing two oligonucleotides (see Additional file 4). The first round of PCR was performed with the primers, STLV-1 Bio5 and Bio4. STLV-1 Bio5 anneals to the sequence within LTR of the STLV-1 provirus and Bio4 is the sequence present in the linker (see Additional file 4). Then, nested PCR was performed with the primers, Ion A-Bio7 and P1. In Ion A-Bio7, uppercase letters denote the sequence that anneals to the viral LTR downstream of STLV-1 Bio5, whereas the sequence in lowercase letters represents a tag specific for the Ion Torrent Personal Genome Machine (Ion PGM). P1 is also a tag specific for Ion PGM, which appears in the linker sequence (see Additional file 4). The amplification conditions of both the first and second PCR were 96°C for 30 sec, 7 cycles of 94°C for 5 sec and 72°C for 1 min, 23 cycles of 94°C for 5 sec and 68°C for 1 min, followed by additional 68°C for 9 min. Amplified fragments of approximately 150–300 bp were size-selected with E-Gel SizeSelect Agarose Gel (Life Technologies) and used as a DNA library in subsequent deep sequencing. Template beads to be sequenced with Ion Torrent Personal Genome Machine (Ion PGM) were prepared with the DNA library using the Ion PGM 200 Xpress Template Kit (Applied Biosystems) and subjected to sequencing on Ion Torrent 314 or 316 semiconductor chip using Ion PGM 200 Sequencing Kit (Applied Biosystems).

### Deep sequencing data analysis

The host genomic sequences, located between the region immediately adjacent to the viral 3’ LTR (ACACA) and the linker sequence (AGATCG), were extracted from the reads. Reads that started with GTTGGG (viral 5’ LTR) were removed. Remaining reads were mapped to the reference genome of *Macaca mulatta* (MMUL 1.0) using the Burrows-Wheeler Aligner (BWA) [53]. Reads that were mapped only to single sites were analyzed. In order to obtain the absolute frequency of each provirus clone (the number of sister cells of the clone), the end position of each mapped read was obtained from the start position and cigar code in the SAM file generated by BWA. The reads with an identical start position and end position (integration site and shear site) were judged to derive from a single DNA fragment amplified by PCR, while reads with identical integration sites but distinct shear sites were judged to derive from different cells in a clone. In other words, the number of reads in the second category reflects the absolute frequency of each clone. Relative frequency represents the proportion of the absolute frequency of a clone to the number of all the sister cells observed. In order to minimize the distortion of relative frequencies of major clones, 6,000 reads that were mapped only to single sites were randomly selected for each specimen and analyzed (see Additional file 2).

### Treatment of STLV-1^+^ Japanese macaques with humanized anti-CCR4 antibody

Two Japanese macaques infected with STLV-1 were treated with mogamulizumab, which is an antibody against CCR4 and is approved in Japan as a drug to treat relapsed ATL. Mogamulizumab was provided by Kyowa Hakko Kirin Co Ltd. One mg/kg mogamulizumab was diluted in 40 ml saline and infused into each monkey intravenously for 20 min. Administration was performed once a week for 4 times. Before each administration, a 10 ml of blood sample was obtained. After the fourth administration, blood samples were collected every 2 weeks until week 11. Extra samples were collected on week 15 and week 18. The two monkeys were observed for any adverse effects during the experiment.

## Competing interests

Kyowa Hakko Kirin provided us the monoclonal antibody (mogamulizumab) that was used in this study.

## Authors’ contributions

JY and M. Matsuoka conceived of this study. JT carried out antibody screening and proviral load measurement. M. Miura, KS, GM and TZ carried out the molecular experiments and the reporter assays. AK, AW, AS and HA coordinated the macaque experiments and collected the macaque specimens. PM analyzed viral protein and surface marker expression. KO carried out immunohistochemistry and pathological analyses. M. Miura carried out massive sequencing and its data analysis. M. Miura, JY and M. Matsuoka prepared the manuscript. All the authors approved the final manuscript.

## Supplementary Material

Additional file 1 Phylogenetic analyses of HTLV-1 subtypes and Japanese macaque STLV-1. Click here for file

Additional file 2 Deep sequencing data analysis. Click here for file

Additional file 3 In vitro staining of Japanese macaque PBMCs with mogamulizumab. Click here for file

Additional file 4 Primers and oligonucleotides. Click here for file
