# Supplementary material for: Characterization of simian T-cell leukemia virus type 1 in naturally infected Japanese macaques as a model of HTLV-1 infection
Source: Retrovirology. 2013 Oct 24;10:118. doi: 10.1186/1742-4690-10-118 (PMC4016002; doi:10.1186/1742-4690-10-118)
Supplement: Additional file 1 — Phylogenetic analyses of HTLV-1 subtypes and Japanese macaque STLV-1. [file 1742-4690-10-118-S1.pdf]

Phylogenetic analyses of HTLV-1 subtypes and Japanese macaque STLV-1

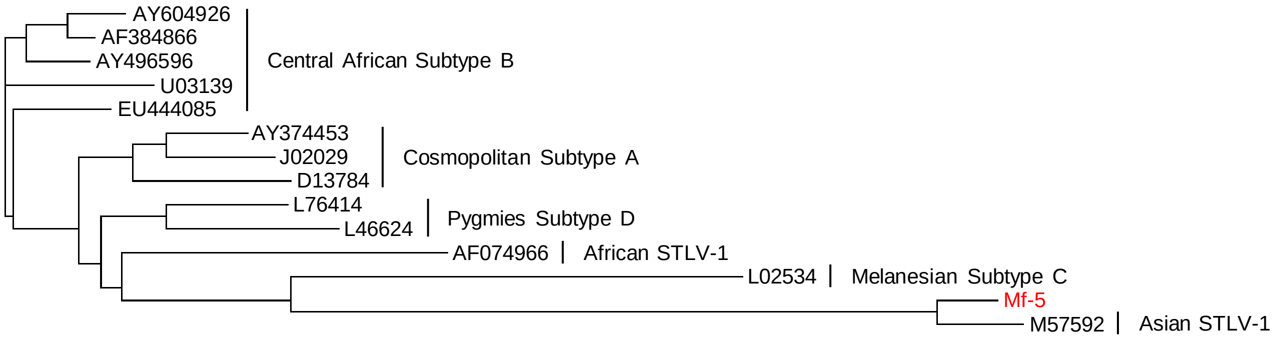

Supplementary Figure 1. Phylogenetic analyses of HTLV-1, STLV-1 subtypes and Japanese macaque STLV-1.

Japanese macaque STLV-1 env region was aligned with exactly the same region (6069-6590 in J02029) of several HTLV-1 and STLV-1 isolates by clustarW (http://www.genome.jp/tools/clustalw/), with default settings. Then, the alignment was processed to generate phylogenetic tree by neighbor-joining method using ClustalW2-phylogeny (http://www.ebi.ac.uk/Tools/phylogeny/clustalw2_phylogeny/) with default settings. Each leaf represents the accession number of the HTLV-1 or STLV-1 isolate.
