# Supplementary material for: Characterization of simian T-cell leukemia virus type 1 in naturally infected Japanese macaques as a model of HTLV-1 infection
Source: Retrovirology. 2013 Oct 24;10:118. doi: 10.1186/1742-4690-10-118 (PMC4016002; doi:10.1186/1742-4690-10-118)
Supplement: Additional file 2 — Deep sequencing data analysis. [file 1742-4690-10-118-S2.pdf]

Deep sequencing data analysis

The length of the mapped reads ranged approximately from 20 to 150 nt (Supplementary Figure 2). This indicates that a clonal abundance (the number of sister cells of a clone in a sample) of up to 130 can be measured by this method, but abundances greater than 130 cannot be measured by this method regardless of how many reads were obtained in a sequencing run. We assessed the number of unique integration sites (i.e. the number of different clones), the number of all the observed sister cells, and the frequency of some abundant clones in the subsamples obtained by randomly selecting certain reads from the total mapped reads (Supplementary Figure 3, left). As is shown in the left column of Supplementary Figure 3, the frequency of abundant clones reached a plateau of around 100, whereas the number of all the observed sister cells continued increasing even at the subsampling size of 10,000 reads. As a consequence, we observed that the relative frequency of each clone declined gradually as we took more reads (Supplementary Figure 3, right). We thus decided to analyze 6,000 reads for each specimen. None of the clones was observed more than 130 times within the subsample of 6,000 reads.

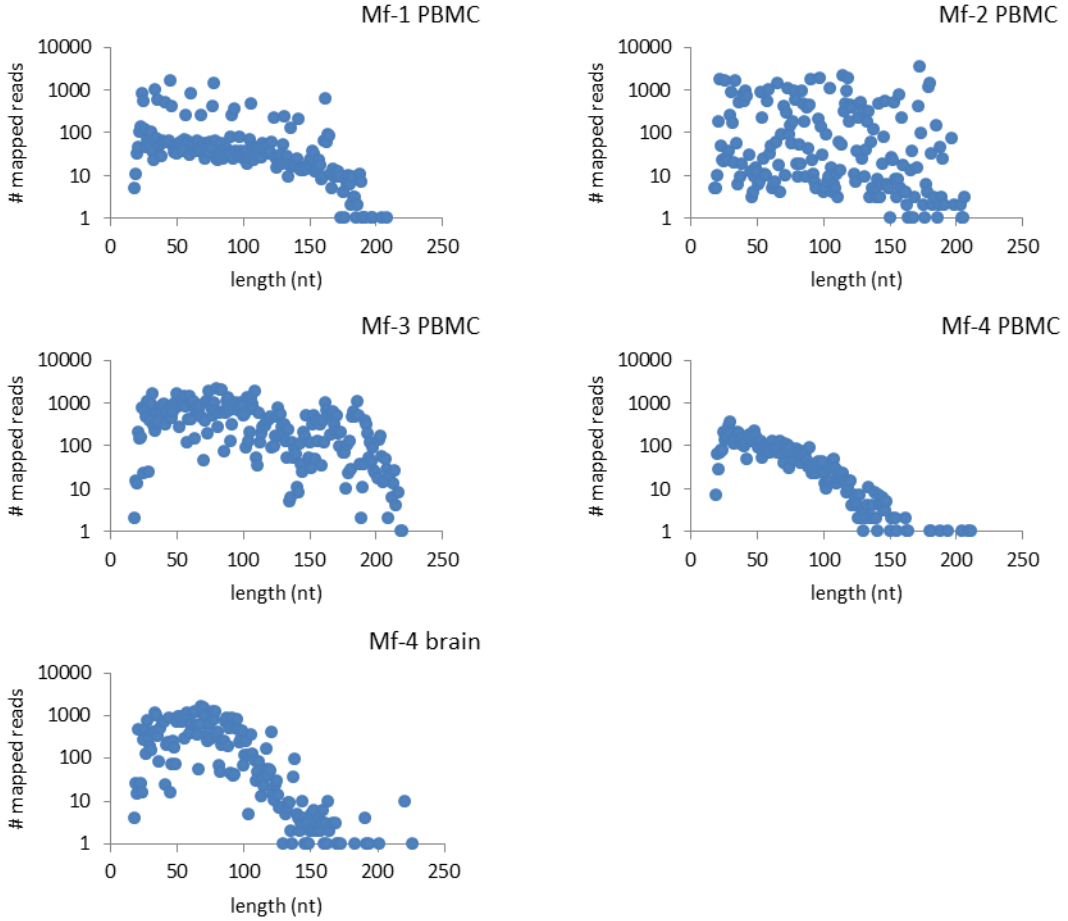

Supplementary Figure 2. Length distribution of reads mapped with single hits.

Sequence flanking the integration site (viral 3' LTR and linker) was removed from each raw read. The trimmed reads were mapped to the reference sequence by BWA (Li H. and Durbin R. 2009. Fast and accurate short read alignment with Burrows-Wheeler Transform. Bioinformatics, 25:1754-60.). The reads mapped to the unique sites were taken and the number of reads of each nucleotide length was plotted.

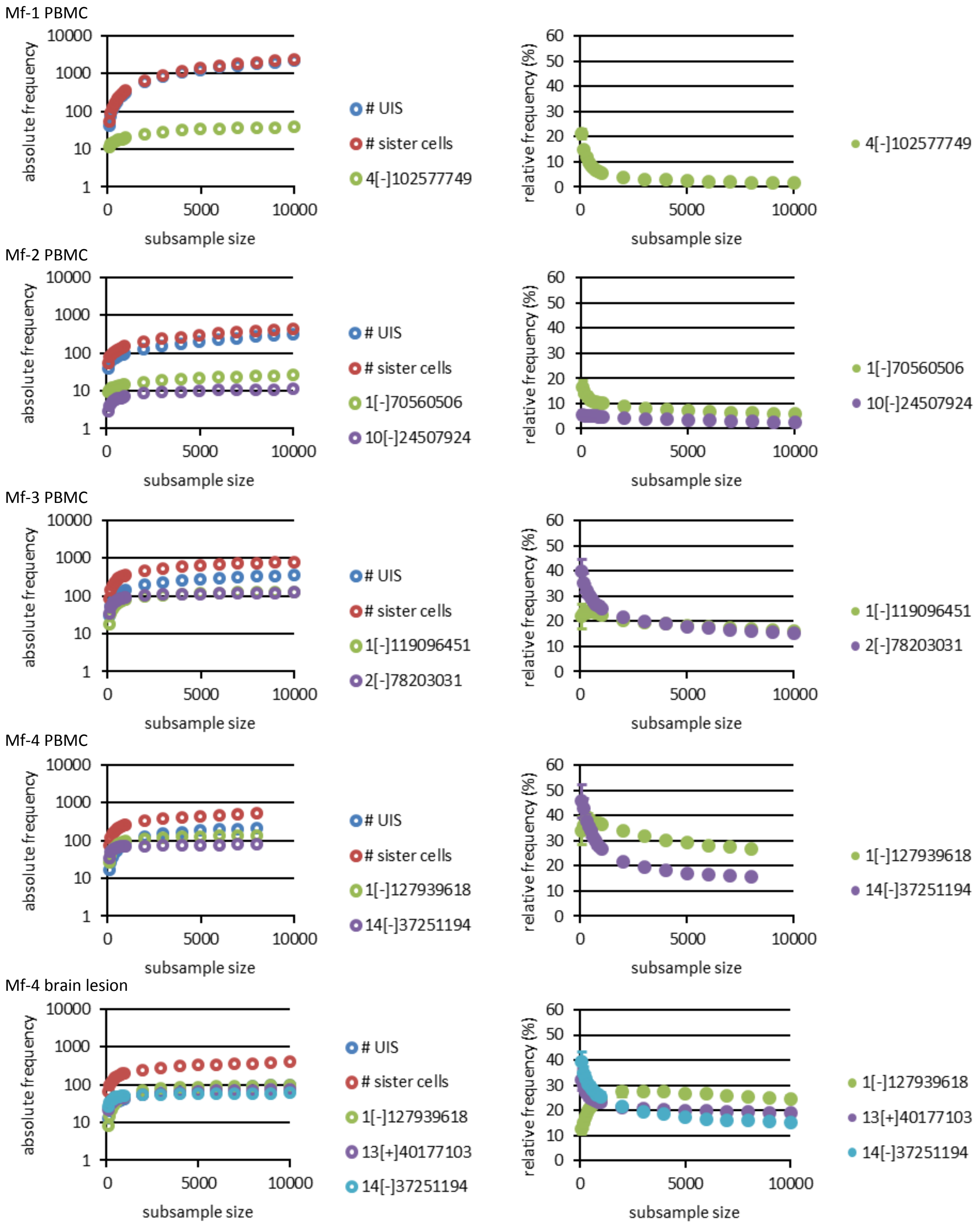

Supplementary Figure 3. Frequency of unique integration sites (UIS), sister cells and some abundant clones.

The mapped reads were randomly selected and the subsamples were generated, then the number of UIS, sister cells and some abundant clones was obtained for each subsample (left). For some of the abundant clones, relative frequency was obtained as the proportion in total sister cells (right). For each subsample size, subsample was obtained six times and the mean value was plotted against each subsample size. In the relative frequency of the abundant clones, standard deviation is also shown.
