# Supplementary material for: Characterization of simian T-cell leukemia virus type 1 in naturally infected Japanese macaques as a model of HTLV-1 infection
Source: Retrovirology. 2013 Oct 24;10:118. doi: 10.1186/1742-4690-10-118 (PMC4016002; doi:10.1186/1742-4690-10-118)
Supplement: Additional file 3 — In vitro staining of Japanese macaque PBMCs with mogamulizumab. [file 1742-4690-10-118-S3.pdf]

In vitro staining of Japanese macaque PBMCs with mogamulizumab

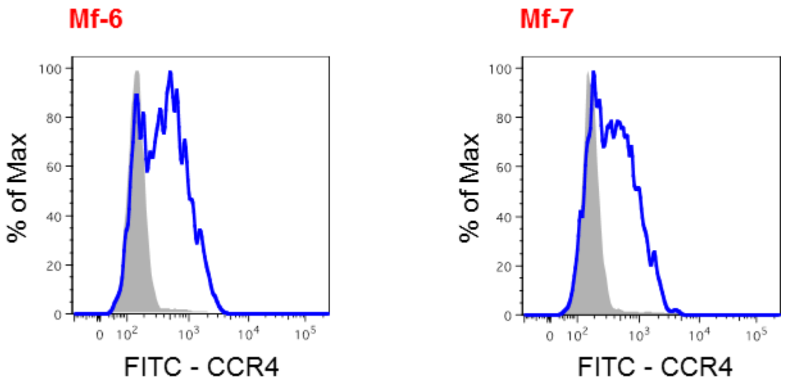

Supplementary Figure 4. Mogamulizumab recognizes macaque CCR4 on the cell surface of STLV-1-infected Japanese macaque PBMCs.

Before we administered mogamulizumab into Japanese macaques, we confirmed that this antibody recognizes not only human CCR4 but also macaque CCR4 on the cellular surface of Japanese macaque PBMCs. We obtained a FITC-labeled antibody which is the same clone as mogamulizumab and stained Japanese macaque PBMCs in vitro. Then, the fluorescence intensity was measured by flow cytometry. The blue line in the histogram shows that mogamulizumab recognizes macaque CCR4. The gray shadow represents the negative control.
