# Supplementary material for: Characterization of simian T-cell leukemia virus type 1 in naturally infected Japanese macaques as a model of HTLV-1 infection
Source: Retrovirology. 2013 Oct 24;10:118. doi: 10.1186/1742-4690-10-118 (PMC4016002; doi:10.1186/1742-4690-10-118)
Supplement: Additional file 4 — Primers and oligonucleotides. [file 1742-4690-10-118-S4.pdf]

Supplementary Table 1. Primers and oligonucleotides used in this study.

Purpose of use	Primer ID	Sequence
Measurement of proviral load	STLV-1 tax_qPCR_F	5′-CTACCCTATTCCAGCCCACTAG-3′
	STLV-1 tax_qPCR_R	5′-CGTGCCATCGGTAAATGTCC-3′
	STLV-1 tax_probe	5′-CACCCGCCACGCTGACAGCCTGGCAA-3′
	Jm RAG1_qPCR_F	5′-CCCACCTTGGGACTCAGTTCT-3′
	Jm RAG1_qPCR_R	5′-CACCCGGAACAGCTTAAATTTC-3′
	Jm RAG1_probe	5′-CCCCAGATGAAATTCAGCACCCATATA-3′
Detection of STLV-1 / HTLV-1 transcripts	tax_forward	5′-CCGGCGCTGCCCTCATTCCGGT-3′
	tax_reverse	5′-GGCCGAACATAGTCCCCCAGAG-3′
	SBZ_forward	5′-CGAACTTACCCAGACGGCGA-3′
	SBZ_reverse	5′-CTGCCGATCGCGATACGTCT-3′
	HBZ_forward	5′-TAAACTTACCTAGACGGCGGACG-3′
	HBZ_reverse	5′-CTGCCGATCACGATGCGTTT-3′
Cloning of expression plasmids	EcoRI-STLV-1 tax_F	5′-CTTAGAATTCATGGCCCACTTCCCAGGTTT-3′
	XhoI-STLV-1 tax_R	5′-GATTCTCGAGTCAGACGTCTGTTTCGCGGA-3′
	EcoRI-SBZ_F	5′-ATCTGAATTCATGGCGGCCTCAGGGCCGTT-3′
	XhoI-SBZ_R	5′-GTTACTCGAGCTACTGCGACCACATTGCTT-3′
	EcoRI-HTLV-1 tax_F	5′-CGAAGAATTCATGGCCCACTTCCCAGGGTT-3′
	XhoI-HTLV-1 tax_R	5′-CTGTCTCGAGTCAGACTTCTGTTTCTCGGA-3′
Amplification of Integration sites	Linker_Long arm	5′-TCATGATCAATGGGACGATCACCTCTCTATGGGCA GTCGGTGATCGCTCTTCCGATCT-3′
	Linker_Short arm	phosphate-5′-GATCGGAAGAGCGAAAAAAAAAAAAA-3′
	STLV-1 Bio5	5′-TGGCTCGGAGCCAGTAGCAGCCCAT-3′
	Bio4	5′-TCATGATCAATGGGACGATCA-3′
	Ion A-Bio7	5′-ccatctcatccctgcgtgtctccgactcag ACTCTCTCCAGGAGAGAGGTTTAGT-3′
	P1	5′-CCTCTCTATGGGCAGTCGGTGAT-3′
